# Transfer of Macronutrients, Micronutrients, and Toxic Elements from Soil to Grapes to White Wines in Uncontaminated Vineyards

**DOI:** 10.3390/ijerph182413271

**Published:** 2021-12-16

**Authors:** Justin B. Richardson, Jahziel K. Chase

**Affiliations:** Department of Geosciences, University of Massachusetts Amherst, Amherst, MA 01003, USA; jahzielchase@umass.edu

**Keywords:** biogeochemistry, plant–soil interactions, wine composition, trace element transfer

## Abstract

Wine is a popular beverage and may be a source of nutrient and toxic elements during human consumption. Here, we explored the variation in nutrient and toxic elements from soils to grape berries and commercial white wines (Chardonnay) at five USA vineyards (New York, Vermont, California, Virginia) with strongly contrasting geology, soils, and climates. Samples were analyzed for macronutrients (Ca, K, and Mg), micronutrients (Mn, Cu, and Zn), and toxic elements (As, Cd, and Pb). Our study showed contrasting macronutrient, micronutrient, and toxic element concentrations in soils and in vines, leaves, and grapes. However, plant tissue concentrations did not correspond with total soil concentrations, suggesting a disconnect governing their accumulation. Bioconcentration factors for soil to grape berry transfer suggest the accumulation of Ca, K and Mg in berries while Fe, Mn, Cu, Zn, and Pb were generally not accumulated in our study or in previous studies. Wines from the five vineyards studied had comparable nutrient, micronutrient, and toxic metal concentrations as wines from Germany, Italy, Portugal, Spain, Croatia, Czech Republic, and Japan. The transfer of nutrients and toxic elements from grape berries to wine indicated that only Ca, K, and Mg were added or retained while concentrations of all other micronutrients and toxic elements were somewhat to extensively diminished. Thus, there appears to be a substantial effect on the geochemistry of the wine from the grape from either the fermentation process (i.e., flocculation), or a dilution effect. We conclude that soils, geology, and climate do not appear to generate a unique geochemical terroir as the transfer and concentration of inorganic nutrients appear to be comparable across strongly contrasting vineyards. This has several implications for human health. Nutrients in wine have potential impacts for human nutrition, as wine can meet or exceed the recommended dietary requirements of Ca, K, Mg, and Fe, and toxic metals As and Pb concentrations were also non-trivial.

## 1. Introduction

Wine is one of the most popular beverages in the world, particularly in the United States. It was estimated that 3.6 gigalitres (966 million gallons) of wine was consumed in the United States in 2018, which corresponds to approximately 11.2 L (2.95 gallons) consumed per resident [1]. Wine is consumed for its complex aromas and flavors, which can stimulate positive emotional links, leading to personal enjoyment [2]. Furthermore, wine is also consumed for positive health benefits, which include lowering the onset of cardiovascular disease, diabetes, osteoporosis and other ailments [3].

Inorganic constituents of wine have garnered interest as a means to examine terroir, the complete natural environment in which a particular wine is produced from grape (*Vitis vinifera*) to bottle. The inorganic constituents can be viewed as a ‘geochemical terroir’, with some of the first geochemical approaches explored by Segiun [4] and Martin [5] to examine potential impacts from geology and soils. More recently, the elemental composition is used to investigate the source of the wine. As a prime example, Galgano et al. [6] demonstrated that wines from three different southern Italian wine-making regions could be distinguished using 29 macro, micro, trace and rare earth elements. Similarly, Cugnetto et al. [7] also found significant differences in the geochemical composition of leaves, grapes, and wines from different regions of northern Italy. Similarly, Coetzee et al. [8] also observed differences in 20 elements in South African wines that appeared to be related to their geographical provenance.

As characterized in an excellent review by van Leeuwen et al. [9], soils have been shown to impact many aspects of vine and grape health and quality, but the mineral chemistry of the soil does not appear to affect the chemistry of the wine. Instead, other aspects of terroir, particularly the human aspects of vineyard management (e.g., Navel and Martins, [10]; Chen et al. [11]) and the viticultural process (e.g., Kristl et al. [12]; Castiñeira et al. [13]) may be more important factors controlling the inorganic constituents within wine.

Exploring the source and transfer for macro and micronutrients from soils to vines to grape berries to wine is important for human health, viticulture, and the validation of wine origin. As previously mentioned, the transfer from soils to wines has implications for daily intake of macro, and especially, micronutrients. Further, the presence of micronutrients such as Fe, Mn, and Cu can affect the wine during the viticultural process as their ability to control oxidation and reduction can influence the formation and catalyzation of acetaldehyde (see [14]). Moreover, excess K can decrease tartaric acid in wines and decrease the pH of grape juice and wine [15]. However, the relationship between the inorganic composition of the wine with the soil is unclear. Roots, root stocks, and specific cultivars can affect the uptake of mineral nutrients from soils, which can control the total or relative abundance of macro and micronutrients [9,16,17]. Furthermore, Kristl et al. [12] and Almeida and Vasconcelos [18] discussed the importance of precipitation reactions during fermentation, settling of colloids, and how contamination from equipment can alter the abundance of micronutrients and trace elements.

Studies examining the macro and micronutrients in vineyards are often limited to one winery or a limited regional samplings of wineries, which use similar viticultural techniques and have comparable terroir factors, such as climate and soil parent material. The primary objective of this study was to quantify macro and micronutrients as well as toxic elements in commercial wines and examine the transfer from soil to vine to grape to wine in strongly contrasting lithologies and climates. In our first hypothesis, we expected differences in the soil and lithology to generate different concentrations in the grape leaves, vines and berries. In our second hypothesis, we expected wines generated across the different soil and lithologies to obtain significantly different nutrient and toxic element concentrations due to differences in the grape berries and vinicultural process. The changes would suggest alternative controls such as the fermentation or filtering process and contamination from equipment could remove or add specific nutrients at greater rates than other elements. This information is needed to inform the transfer of elements from soils to vines to grapes to wines and explore its ramifications for health benefits of wine.

## 2. Materials and Methods

### 2.1. Descriptions of Vineyard Studied

**Figure 1:**
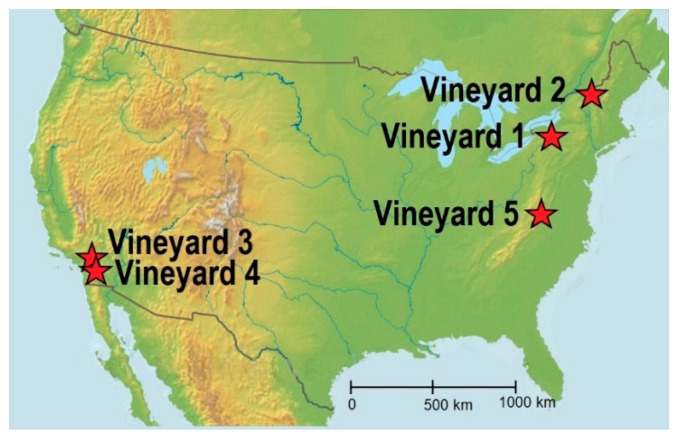
Location of vineyards investigated in this study.

We studied five conventional vineyards across North America (Figure 1), with strongly contrasting climates and soils. Due to standing agreements with each of the vineyards, the authors are obligated not to release their names and specifics about their agricultural systems. However, generalized descriptions are provided below for each of the vineyards and soil data are from the USDA-NRCS [19]. All vineyards have been in production for over 15 years, vines are vertically positioned, trained, and bilateral cordon spur pruned and alleys were 2–2.5 m apart. At all the vineyards, grape pomace, composted plant materials, synthetic soil amendments are used and synthetic insecticides are used. Vineyard 1 is situated along a hill slope in the Finger Lake Region of New York State, USA (Table 1). Vineyard 1 has a humid continental climate, is underlain with grey shale bedrock and soils are formed from primarily shale-derived glacial till with minor inclusions of granite and schists. Rootstocks used were *Millardet and de Grasset 420A* and *Richter 99.* Vineyard 2 is located on a glacial-lake plain in the Champlain valley of northwestern Vermont. Vineyard 2 has a humid continental climate, is underlain with argillite and soils are formed from lacustrine clay-rich deposits. Rootstocks used were Vineyard 3 is located on a terrace in the Temecula Valley of southern California. Due to the clay-rich soil horizons, the soils have a higher seasonal water compared with the other locations. Vineyard 3 has a hot-summer Mediterranean climate, underlain with granite and soils are formed from granitic residuum. Vineyard 4 is located on an alluvial fan in northern San Diego County, of southern California. Vineyard 4 also has a hot-summer Mediterranean climate, but soils are formed from granitic alluvium. Vineyards 3 and 4 are irrigated with microdrip irrigation. Rootstocks used in Vineyards 3 and 4 include 110 Richter, 1103 Paulsen, and 11103P. Vineyard 5 is located on a footslope along the Blue Ridge of the Shenandoah Valley of central Virginia. Vineyard 5 has a humid subtropical climate, is underlain with granite but soils are formed from granitic colluvium and highly weathered granitic residuum.

### 2.2. Soil Sampling and Physicochemical Analyses

At each vineyard, one field (1–3 ha) was sampled for soils, vines, leaves, and grapes. Nine soil pits were sampled by excavating to ~0.5 m depth to collect 1 kg samples of the master A and B horizons across each of the five vineyards in September–November of 2018 for Vineyards 1 and 2 and 2019 for Vineyards 3, 4, and 5. Soil pits were distributed evenly across the fields, with an emphasis on observing variations within the vineyards. Soil pits were excavated to capture A and B horizons, which were approximately 30 to 40 cm in depth. Soils were oven dried at 50 °C for 72 h, sieved to <2 mm, and homogenized for analysis.

Soils were characterized for their basic physicochemical properties. To determine soil pH, a 2:5 soil−water slurry was used. Slurries were shaken for 1 h and filtered through a Whatman 40 filter. The pH of extract was measured with a pH meter (8015 VWR). Loss on ignition was used to estimate % soil organic matter (SOM) and measured by combusting a 4 g oven-dried subsample at 550 °C for 8 h. Every 20 samples included one blank and duplicate. California soils were treated with nitric acid first to remove carbonates. To determine the soil particle size distribution, a modified Bouyoucos hydrometer method was used [20]. First, 30.0 ± 2.0 g of dried soil was weighed into 250 mL glass beaker and dispersed with 100 mL of 1 M sodium hexametaphosphate (HMP) for at least 8 h. The HMP-soil slurry was washed out into a 1000 mL graduated cylinder with DI water, shaken, and hydrometer readings were taken at 60 s and 1.5 h after mixing to the closest 0.5 g/L.

A soil subsample was digested for total elemental concentrations, which quantifies nutrients complexed to organic matter and clays, within oxides, and within silicate minerals using an HNO_3_-HF total digestion method. A 50 mg homogeneous subsample of each soil horizon was placed in a 30 mL PFA vial with 2.5 mL of 70% HNO_3_ and 2.5 mL of 35% HF and heated to 170 °C for 48 h in triplicate. The digest was dried to a moist paste and re-suspended with 2 mL of HNO_3_ and dried down again to a paste. The final paste was digested with 5 mL of 35% HNO_3_ at 170 °C for 48 h and diluted to 50 mL with DI water. Every total soil digest batch of 25 samples included one preparation blank and standard reference material NIST 2709a San Joaquin Soil.

### 2.3. Vine, Leaf, Grape, and Wine Collection and Digestion

To sample grape plants, two plants near each of the nine soil pit locations (18 plants in total) were selected and sampled near or at the end harvesting season September 2018 for Vineyards 1 and 2, and October 2019 for Vineyards 3 and 4, and September 2019 for Vineyard 5. The end of the season was chosen to capture the total amount of macro and micronutrients acquired by each structure and concentrations in the berries that will be used in the viticulture. To sample vines, three cane segments (10 cm in length and 1 to 2 cm diameter) from the end of the growing season were collected. To determine leaf uptake, 6 to 8 mature leaves, lacking any visual signs of chlorosis, infection, discoloration or edge burning were collected. Lastly, three bunches of grapes were collected from each plant of the 18 total plants. Grapes were within 2 weeks of their harvest date. Cane segments and leaves were oven dried in paper bags at 50 °C for 48 h. Grapes were removed from the pedicel, frozen at −40 °C, crushed while frozen, and freeze-dried to a constant weight. Oven drying is not recommended as it can form recalcitrant organic compounds within the berries.

To determine macro and micronutrient concentration digestions within plants, a total digestion was performed based upon the EPA 3050B Method [21,22], in which samples are combusted prior and digested with strong acids. First, vine segment bark was removed and leaf blades were separated from mid-veins and petioles. Vine segments, leaf blades, and freeze-dried berries were crushed with a mortar and pestle or ground cut with stainless steel blades to <0.5 mm and the fragments were further ground to <0.1 mm with a manual coffee grinder with stainless steel mill blades. Next, ground material was transferred to ceramic vessels and combusted at 550 °C for 8 h. The ashes were transferred to 50 mL centrifuge tubes and digested with 5 mL of reverse aqua regia (9:1 Trace Metal Grade HNO_3_:HCl) and heated to 90 °C for 45 min. After 12 h, the digest was diluted to 50 g using deionized water. Every extraction and digest batch of 25 samples included one preparation blank, one duplicate, and standard reference material NIST 1547 Peach Leaves.

We purchased three Chardonnay white wines made with grape berries from only the vineyards studied using the previous years vintage. Wines were digested for analysis for their macro and micronutrient concentrations. Only white wines were selected that utilized grapes from the field sampled from the previous year’s vintage. To digest the wine samples, 200–300 mL of the wine was heated to 100 °C in a glass beaker and treated with 70 to 150 mL of 30% H_2_O_2_ to oxidize sugars, phenolics, ethanol, and other dissolved organic carbon compounds to CO_2_. After the solution became clear and excess H_2_O_2_ was detected via H_2_ and O_2_ breakdown products, the wine digest was concentrated to 30 mL and acidified with 2 mL of 15.8 M HNO_3_ to keep metals in solution. For elemental analysis, 3.0 ± 0.1 g of digest was diluted to 12.0 ± 0.1 g with 0.45 M HNO_3_.

### 2.4. Elemental Analyses

Soil extracts, soil digests, plant tissue digests, and wine digests were analyzed for macronutrients (Ca, K, and Mg) with an Agilent 5110 Inductively Coupled Plasma-Optical Emission Spectrometer (Agilent Technologies, Santa Clara, CA, USA). Total digestion of San Joaquin SRM 2709a, total digestions of Peach Leaves SRM 1547a, and spiked wine samples had recoveries for Ca, K, Fe, and Mg were 81–112% of their certified values. Nutrient concentration coefficient of variation between intra-sample duplicates was <11% for total soil digestions and <6% for plants and wines digests. Metal concentrations in the preparation blank samples were <0.2% of their analyte concentrations.

Trace elements (As, Cd, Cu, Mn, Pb, and Zn) were determined with an Agilent 7700x Inductively Coupled Plasma Mass Spectrometer (Agilent Technology, Santa Clara, CA, USA). Total digestion of San Joaquin SRM 2709a, Peach Leaves SRM 1547a, and spiked wine samples recoveries for As, Cd, Cu, Mn, Pb, and Zn were 82–111% of their certified values. Micronutrient coefficient of variation between intra-sample duplicates was <9% for total soil digestions and <5% for plants and wines digests. Micronutrient and toxic element concentrations in the preparation blank samples were <0.1% of their analyte concentrations.

### 2.5. Statistical and Data Analyses

Descriptive statistics were calculated in Matlab (Mathworks, Natick, MA, USA). Average values are presented in text and in figures ±1 standard error. Non-parametric statistical tests (Kruskal–Wallis test with post hoc Wilcoxon Rank Sign test) were used to compare difference among plant-available soil, total soil, vine, leaves, grapes, and wine macro and micronutrient concentrations and also soil properties (pH, %SOM). Data were log transformed to avoid biases incurred using assumptions and descriptive statistics for normally distributed data.

To determine elemental transfer from soil to grape, bioconcentration factors (BF) were calculated as the ratio of grape berry concentrations (µmol/g) were divided by the in soil concentrations in (µmol/g) (Equation (1)). Similarly, the movements of elements from grape berries to wine was calculated as transfer coefficients, which is the ratio of wine concentrations (µmol/g) were divided by the grape berry concentrations in (µmol/g) (Equation (2)).

To visualize these ratios, they were logarithmically transformed and plotted.

(1) $$\mathrm{Soil}\;\mathrm{to}\;\mathrm{grape}\;\mathrm{berry}\;\mathrm{Bioconcentration}\;\mathrm{factors}=\frac{\left[Grape\;berry\right]}{\left[Total\;Soil\right]}$$

(2) $$\mathrm{Grape}\;\mathrm{berry}\;\mathrm{to}\;\mathrm{white}\;\mathrm{wine}\;\mathrm{transfer}\;\mathrm{factors}=\frac{\left[white\;Wine\right]}{\left[Grape\;berry\right]}$$

## 3. Results

### 3.1. Soil Physicochemical and Nutrient Concentrations

Soil physicochemical properties of exhibited a wide range in values for %SOM and particle size distribution, but pH was relatively similar across vineyards. Soil pH was most acidic in the B horizons for Vineyard 5 at pH 5.04 (Table 2), which was greatly expected due to the soil being much older, with fewer primary minerals remaining and low base cation availability [23]. Soil pH was high in Vineyards 3 and 5, with a pH of 6.3, which was also expected due to higher MAT and semi-arid nature of southern California. However, soil pH was highest in Vineyard 2 in Vermont, which is most likely due to the calcareous nature of the argillite and sedimentary bedrock present [19]. Soil pH was similar to soils in vineyards in France [24], Italy [25] Portugal [26], and Greece [27]. Soil organic matter was largely comparable across sites, typically with higher %SOM in A horizons (6.2–10.7%), except for Vineyard 4, which only had an average of 4.3% SOM. B horizons had a lower %SOM, ranging from 4.1 to 8.2% SOM, except for Vineyard 4, which only had an average of 2.8% SOM. The higher %SOM in the A horizons can be attributed to the application of composted plant matter as part of the management strategy.

Total soil concentrations of macro and micronutrients show some substantial differences across the five vineyards (Table 3) particularly in Vineyard 5, but are within ranges reported from other vineyards in in France [24], Italy [25] Portugal [26], Greece [27], Spain [28], and Serbia [29]. Vineyard 5 had significantly lower total concentrations of Ca, K, Mg, and Mn, which can be attributed to extensive loss of primary minerals in the Ultisols present, e.g., [23]. Total soil Ca concentrations were largely comparable among Vineyards 1, 3, and 4 (Table 3). Total soil Ca concentrations were significantly higher for Vineyard 2 than the other vineyards (*p* < 0.05), coinciding with the highest soil pH and further demonstrating the effect of the calcareous soil parent material. Total soil K concentrations were significantly higher for Vineyard 3 (*p* < 0.05), but comparable among Vineyards 1, 2, and 4. Total soil Mg concentrations were comparable for Vineyards 1 and 2 but were significantly lower than for Vineyards 3 and 4 (*p* < 0.05) (Table 3). Vineyard 5 had the lowest Ca, K, and Mg due to the extensively weathered A and B horizons, as it is the wettest of the climates. Differences in K and Mg between the two California vineyards, Vineyards 3 and 4, illustrate differences in the geologic materials and management can generate significant differences despite a being in the same climate.

There were also significant differences for micronutrient and toxic elements in soils. Total soil Fe were comparable for Vineyards 1, 2, 3, and 4 but significantly higher for Vineyard 5 (*p* < 0.05), but has higher concentrations of Fe due to formation of secondary Fe oxides. This agrees with previous studies that observed more abundant Fe oxides in vineyards in soils with Ultic B horizons, in which primary minerals are highly weathered and Al and Fe minerals dominate [23,30]. Total Mn was comparable across Vineyards 1, 2, 3, and 4 but was significantly lower for Vineyard 5 (*p* < 0.05) (Table 3). Total soil Cu were comparable for Vineyards 2, 3, and 4. Vineyards 1 and 5 had significantly lower total soil Cu than the other vineyards. Total soil Zn was significantly lower in Vineyards 1, 2, and 5 than Vineyards 3 and 4 in California. Total As, Cd, and Pb in Vineyards 1 and 2 were significantly higher than Vineyards 3 and 4 in California.

However, the variation in the total micronutrient concentrations of these micronutrients is most likely due to geological and pedological differences across the five vineyards [4,9,31]. Soil Cd and Pb were significantly higher in Vineyard 1 than all other vineyards despite being a rural, agricultural area located away from local pollution sources. However, Cd and Pb in the soil can be inherited from the grey shale present (e.g., see trace elements in carbonates such as Protano and Rossi [25] or grey shales in Richardson et al. [32]. Vineyard 5 had comparable As concentrations and Vineyards 3 and 4, but had significantly higher Pb and lower Cd than Vineyards 3 and 4. The higher Pb is likely due to recent long-range transport and deposition or addition, as Pb should be depleted from the extensive weathering [32], as observed with the lower Cd and Cu concentrations in Vineyard 5.

In addition to soil and lithological affects, differences among the vineyards in micronutrient concentrations can be due to soil management techniques. For example, Cu is commonly used against fungal diseases and is sprayed onto whole plants [27]. Non-trivial concentrations of Zn and Pb could be substituted into Cu plant fungal treatments and Cd, Zn, and Pb can be added to soils within lime fertilizers [33]. This could explain the elevated Zn and Pb in Vineyard 5. Furthermore, As and Cu can be sourced to vineyard soils through decomposition and leaching from CCA treated wood used as posts for positioning canes [34].

### 3.2. Vine, Leaf, and Grape Berry Nutrient and Toxic Element Concentrations

Most of the vine, leaf, and grape berry macronutrient concentrations were significantly different across the five vineyards. Vines exhibited significant differences among the five vineyards for all macronutrients, micronutrients, and toxic elements, except Ca and Mg. Total vine concentrations of Ca and Mg were largely comparable across the vineyards, ranging from 2.0 to 4.6 g/kg Ca and 0.6 to 2.1 g/kg Mg (Table 4). Total vine concentrations of K were not different for Vineyards 1, 2, and 5 but were significantly lower in Vineyards 3 and 4. Total vine Fe concentrations were similar for Vineyards 1, 2, and 5 but were significantly greater for Vineyards 3 and 4 (Table 4). Total vine Mn, Cu, and Zn concentration exhibited a wide range of concentrations, typically with highest concentrations in Vineyard 5 and lowest in Vineyard 1. For toxic metals, Vine As and Cd were lowest for Vineyard 1 and highest for Vineyard 4 and 5. Vine Pb concentration were significantly greater for Vineyards 1 and 2 than Vineyards 3 and 4.

Leaves exhibited significant differences among the five vineyards for all macronutrients, micronutrients, and toxic elements, except Mg. Total leaf Mg concentrations were largely comparable across the vineyards (Table 4). Total leaf concentrations of Ca, Fe, and Mn were significantly higher for Vineyards 3 and 4 than Vineyards 1 and 2 (*p* < 0.05) (Table 4). Conversely, total leaf K was significantly lower for Vineyards 3 and 4 than Vineyards 1 and 2 (*p* < 0.05). For total leaf Cu and Zn, concentration were significantly higher in Vineyard 5, comparable for Vineyards 2, 3, and 4. For toxic elements, As, Cd, and Pb were generally significantly higher for Vineyards 2, 3, and 4 than Vineyards 1 and 5 (Table 4). Leaf Ca, K, Mg, Fe, Mn, Cr, and Zn concentrations in our study were comparable with values reported by Angelova et al. [35], Chopin et al. [24], Cugnetto et al. [7], Milićević et al. [29], Vystavna et al. [36], and Vystavna et al. [37].

Total grape berry concentrations of Ca, Mg, and Fe were significantly higher for Vineyards 3 and 4 than Vineyards 1, 2, and 5 (*p* < 0.05) (Table 4). Total grape berry concentrations of K were significantly higher for Vineyards 3 and 4 than Vineyards 2 and 5 (*p* < 0.05). Total grape berry concentrations of K were significantly higher for Vineyards 3 and 4 than Vineyards 2 and 5 (*p* < 0.05). For micronutrients, total grape berry concentrations of Mn and Cu were significantly higher for Vineyards 3, 4, and 5 than Vineyards 1 and 2 (*p* < 0.05) (Table 4). Total grape berry Zn concentrations were greatest for Vineyard 5 and lowest for Vineyard 1. For toxic elements, Vineyards 2 had significantly higher As and Cd concentrations than Vineyards 1 and 5 (*p* < 0.05) (Table 4), while grape berry Pb concentrations were greatest for Vineyard 2 and lowest for Vineyard 5. Grape berry Ca, K, Mg, Mn, Cr, Cu, and Zn concentrations in our study were within the range of values reported by Angelova et al. [35], Cabrera-Vique et al. [38], Castiñeira et al. [13], Catarino et al. [26], Chopin et al. [24], Cugnetto et al. [7], Milićević et al. [29], Pepi et al. [28], Protano and Rossi. [25], Vystavna et al. [36], and Vystavna et al. [37].

Our vine, leaf, and grape berry data demonstrate some consistency with macronutrients to obtain the biochemical stoichiometry needed by Vitis vinifera but our results demonstrate that micronutrient and toxic element uptake varies significantly, in some cases over an order of magnitude across the vineyards. However, due to the co-varying factors of climate, geologic materials, soils, and management practices, it is difficult to discern which variables led to higher nutrients and toxic elements. Generally, leaves and grape berries had higher Ca and Mg concentrations in Vineyards 3 and 4 than the other vineyards, which did not necessarily correspond with higher soil Ca and Mg. Thus, it appears climatic and management factors have affected these two macronutrients. For example, irrigation could have added soluble Ca and Mg to the soils or allowed for higher uptake compared with plant materials in non-irrigated areas. For Fe and Mn, their abundance in soil also did relate to higher leaf and grape berry concentrations. To the contrary, soil Fe was highest in Vineyard 5 but leaf and grape berry concentrations were higher for Vineyards 3 and 4. Further, soil Mn was highest in Vineyard 1 but leaf and grape berry concentrations were higher for Vineyards 3 and 4. This further suggests that lithology and soil likely did not control Mn and Fe in the fruit. For toxic elements, vine, leaves, and grape berry concentrations of As, Cd and Pb were largely similar except for vine, leaves, and grape berries from Vineyard 1, despite having higher soil As, Cd, and Pb concentrations. This implies either the soil physicochemical properties, plant ecophysiology, or management practice has decreased As, Cd, and Pb uptake in Vineyard 1. For example, root stock species and cultivars can control the uptake of soil nutrients, with the potential generate chemical characteristics different from the soil [16]. Moreover, Coombe [39] found evidence that inflow of phloem sap and isolation from vascular transport can affect physical and chemical characteristics of grape berries. Further, translocation of solutes into the berry can be regulated by phloem movements [40], which can control the accumulation of micronutrient and toxic elements regulate of multiple growing seasons [17]. These results suggests that the geochemical and biochemical concentrations in aboveground tissues had minimal connection to soil abundance of macronutrient and toxic element concentrations.

### 3.3. Wine Nutrient and Toxic Element Concentrations

When examining macronutrient, micronutrient, and toxic element concentrations in white wines from the five vineyards, most elements exhibited moderate variations, of a factor of 0.5 up to 2.0. Ca and Mg concentrations were similar in concentration, varying from 61 to 106 mg/kg for Ca and 77 to 120 mg/kg for Mg (Table 5). Similarities across vineyards continued with wine Cu, As, and Cd, which varied from and 11 to 23 µg/kg for Cu, 3.3 to 5.5 µg/kg for As, and 0.09 to 2.3 µg/kg for Cd. There were substantial variations in wine K, Fe, Mn, Zn, and Pb concentrations, on a factor of 3 to >10. Wine K concentrations were significantly higher in Vineyards 3 and 4 (*p* < 0.05) (Table 5). Wine Fe concentrations were significantly higher in Vineyard 1 than the other vineyards while Vineyards 2 and 5 had significantly lower wine Fe concentrations (*p* < 0.05) (Table 5). Wine Mn concentrations were comparable across Vineyards 1, 2, 3, and 4 but were significantly higher in Vineyard 5 (*p* < 0.05) (Table 5). Wine Zn concentrations were highest in Vineyard 5, comparable between Vineyards 3 and 4, and lowest in Vineyard 2. Wine Pb concentrations were comparable for Vineyards 2, 4, and 5 but were significantly higher in Vineyards 1 and 3 (*p* < 0.05) (Table 5).

Wine concentrations were largely comparable to other macro and micronutrient concentrations from other countries. Wine Ca, K, Mg, and Mn concentrations were comparable to concentrations in red and white wines from Germany, Italy, Portugal, Spain, Croatia, Czech Republic, and Japan (Table 5). Wines concentrations of Fe, Cu, and Zn in our study appear to be lower than wines in other regions. For example, wine Fe concentrations in our study ranged between 0.3 to 2.4 mg/kg but were as high as 4.6 mg/kg in Castiñeira et al. [13] and 5 mg/kg in Gonzálvez et al. [41]. Furthermore, wine Cu concentrations were <25 µg/kg but exceeded 400 µg/kg for Catarino et al. [26], Kment et al. [42] and Vrček et al. [43]. Similarly, wine Zn concentrations were <600 µg/kg but exceeded 1000 µg/kg in Castiñeira et al. [13], Hopfer et al. [44], and exceeded 2000 µg/kg in Vrček et al. [43]. Wine As concentrations in our study were comparable with many other studies, which had As concentrations ranging from 0.9 up to 6.7 µg/kg [13,26,43,45]. Wine Cd concentrations in our study, which ranged from 0.09 to 2.3 µg/kg, were lower than other studies, Leder et al. [45] and Vrček et al. [43]. Lastly, wine Pb concentrations in our study were comparable with studies across the world, ranging from 1.2 up to 67 µg/kg [13,26,43,45,46].

### 3.4. Transfer of Nutrients and Toxic Elements from Soil to Grapes and Grapes to Wine

**Figure 2:**
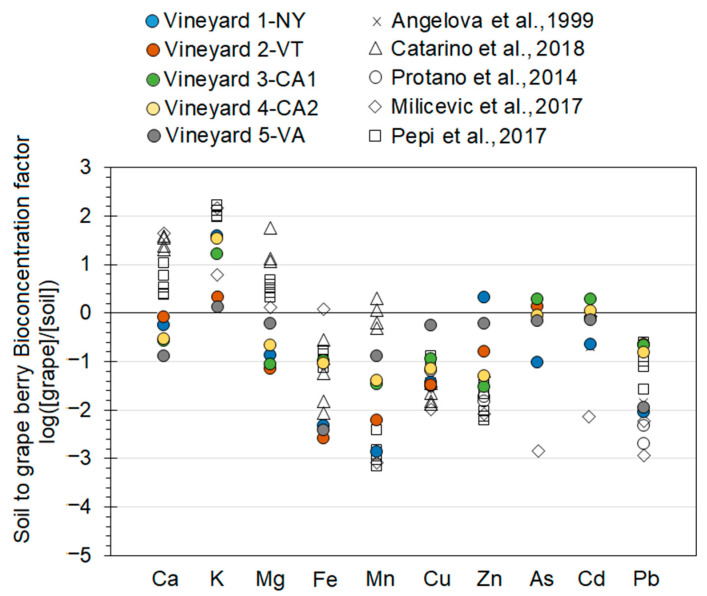
Bioconcentration factors (BF) for macronutrients, micronutrients and trace elements from soils (total digestion) to grapes berries at the five vineyards studied and five previous studies are plotted on a logarithmic axis.

We calculated bioconcentration factors (BF) for macro and micronutrients and toxic elements from soils to grape berries (Equation (1)), which were logarithmically transformed and plotted in Figure 2. A log-transformed BF values less than 0 denotes soil concentrations were higher than grape berry concentrations, implying active exclusion or inability of uptake to the grape berry. Conversely, log-transformed BF values greater than 0 denote soil concentrations were lower than grape berry concentrations, implying accumulation in the grape berry. For macronutrients in our study, log-transformed BF values for Ca, K, and Mg were between −1 and 2.3 (Figure 2), suggesting minimal exclusion with one or two orders of magnitude of accumulation/addition to grape berries. This shows that grapes are enriched in macronutrients compared to soils. This agrees with the general change from macronutrients diluted by Si and other rock forming inorganic elements in soils to C and other plant forming organic elements. Further it notes that plant tissues accumulated these elements to meet nutritional demands. It is important to note that the log-transformed BF values are higher in other previous studies by Angelova et al. [35], Catarino et al. [26], Protano and Rossi [25], Milićević et al. [29], and Pepi et al. [28], largely due to their use of nitric and hydrochloric pseudototal digestions of soils as opposed to our hydrofluoric acid based total digestion. Thus, our more effective digestion of soils able to digest silicate minerals produced higher measured soil concentrations [21], which would produce lower BF values. Log-transformed BF values for micronutrients and toxic elements were <0, indicating limited accumulation to restricted uptake from soil to grape berries (Figure 2). This can either be due to their abundant concentrations in soils compared with uptake to grape berries (e.g., Fe, Mn in particular), discrimination in root uptake and shoot translocation (e.g., Cu, Zn, and Pb), or were generally taken up at a comparable rate with their presence in soils (As and Cd), which match observations from previous studies. A possibility for higher BF values for As and Cd than other macro- and micronutrients is the uptake of As instead of P or Cd uptake instead of Ca [47,48]. Our results highlight that macronutrients (Ca, K, and Mg) and some toxic elements (As, Cd) were accumulated or had minor limitations in uptake and translocation from soil to grape berries across lithology, climate, and management practices. Uptake and translocation of micronutrients such as Fe, Mn, Cu, Zn, and Pb were discriminated against with a wide range in uptake and translocation rates, spanning nearly two to three orders of magnitude. These elements are likely more sensitive to lithology, climate, and management practices.

**Figure 3:**
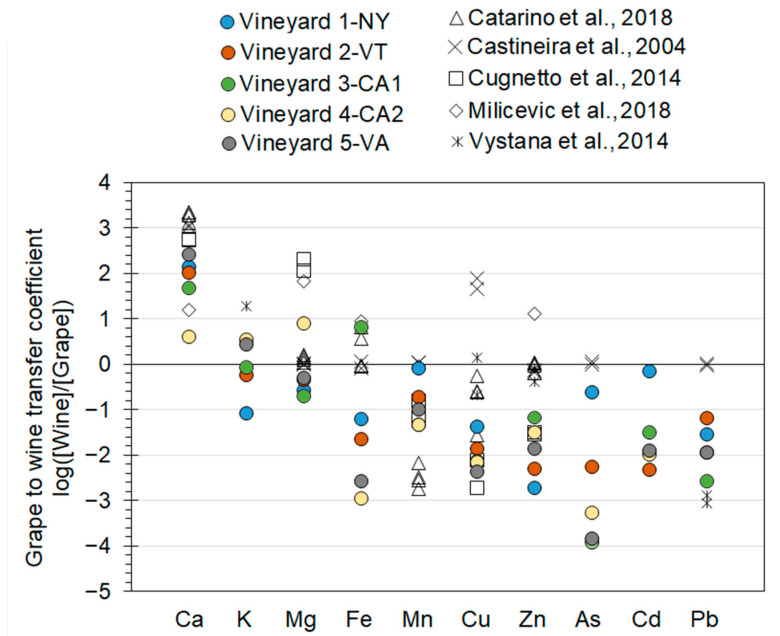
Transfer coefficients of macronutrients, micronutrients, and trace elements from grape berries to white wines at the five vineyards studied and five previous studies are plotted on a logarithmic axis.

We also calculated grape berry to wine transfer coefficients to determine their transfer efficiency during the viticulture process (Equation (2), Figure 3). Log-transformed transfer values <0 indicate limited to extensive reduction in transfer from grapes to wine while values >0 implies limited to high transfer or addition to wines relative to grapes. Log-transformed transfer coefficient values for Ca, K and Mg show enrichment to limited restrictions from grape berries to wine (Figure 3), indicating either an efficient transfer or addition during the viticulture process. White wines are commonly treated with cream of tartar (KC_4_H_5_O_6_), bentonite, gelatin, alum, or lime (CaO) as flocculants to remove suspended particles and improve clarity, which can also add macronutrients and other metals to the wine [49]. Nearly all micronutrients and toxic elements had transfer coefficients less than 0, indicating some to extensive reduction in transfer from grapes to wine (Figure 3). Transfer coefficients for Mn Cu, Zn, As, Cd, and Pb were <0 (Figure 3), suggesting exclusion/removal from grape to wine during the viticulture process. This agrees with Almeida and Vasconcelos (2003) that redox sensitive elements such as Fe or Mn can be removed during the fermentation process by either precipitation (potentially FeS_2_) or complexation and sedimentation on organic colloids [50], particularly with the addition of flocculants mentioned earlier. Based upon decreasing in micronutrient and toxic element concentrations, our findings disagree with previous findings by Kristl et al. [12] and Almeida and Vasconcelos [18], who found that micronutrients and toxic elements are added as grapes are fermented to grapes through contamination by processing equipment. Although we did not see an effect on the transfer ratio, we do not have the data to confirm no metals were added from the wooden barrels, stainless steel equipment, or other processing equipment. Furthermore, a dilution effects are not captured by investing concentrations and masses and volumes are needed to determine changes in overall element additions or removals. The volume of juices extracted from the grape berries can decrease due to evaporation from differences in air humidity but water can be added during the juicing stages [51]. With the addition of water, it can decrease measured concentrations and thus lower transfer coefficients measured. Unfortunately, this information was not available to us from the five vineyards studied but as macronutrient (Ca, K, and Mg) and even some micronutrients (e.g., Mn) did not show consistent decreases in concentrations, it suggests dilution and concentration effects are likely to be limited and not the driver for lower micronutrient and toxic metal concentrations in the wine. Further studies are needed to determine if there are additional sources adding metals. Mass-balance techniques can be applied as well as more advanced geochemical techniques such as the application of stable isotopes to effectively constrain provenance of nutrients and toxic elements.

### 3.5. Implications for Wine Macro and Micronutrient Concentration on Human Health

To relate our findings to human health, we calculated the daily percentage of macro and micronutrients ingested per glass of wine. The density of a 14% alcohol white wine (typical for Chardonnay) was measured to be 0.994 g/mL at 20 °C by Kunkee and Eschnauer [52]. Next, we referenced the standard pour for a glass of wine in the U.S., which is 5 fluid ounces or 148 mL and calculated the mass of nutrients in a glass of wine using our lowest and highest macro and micronutrients concentrations observed in the wines in our study. The nutrients in a glass of wine were compared to United States National Institute of Health (NIH) Recommended Dietary Allowance (RDA), which is an estimate of the average daily level of intake sufficient to meet the nutrient requirements of nearly all (97%) healthy people, based on the reference caloric intake of 2000 kcal for adults [53]. The NIH RDA values are the following: Ca 1300 mg/day, K 4700 mg/day, Mg 420 mg/day, Fe 18 mg/day, Mn 2.3 mg/day, Cu 0.9 mg/day, and Zn 11 mg/day (NIH Office of Dietary Supplements, 2020).

We determined the percentage of the total daily RDAs for a single glass of white wine are as follows: for Ca 69% to 120%, K 165% to 496%, Mg 270% to 420%, Fe 25% to 196%, Mn 448% to 1791%, Cr 126% to 206%, Cu 1.8% to 3.8%, and Zn 0.66% to 0.73%. Thus, a single glass of white wine could represent more than the daily RDA for K and Mg and depending on the wine, Ca and Fe as well. The abundance of Cu and Zn in wines was well below RDA. Thus, wine can supply metal nutrients vital for normal body functioning and help avoid deficiencies [53,54]. Conversely, white wine from our study would result in consuming 0.5 to 0.8 µg per glass of As, 0.01 to 0.03 µg per glass of Cd, and 0.5 to 2.2 µg per glass of Pb. Arsenic was well below the 10 µg/L drinking water standard and humans typically consume far more water per day (3.7 L/day) than wine. Similarly, Cd concentrations are far below United States EPA drinking water standard of 5 µg/L and also far below the 1 µg per kg of body weight per day, assuming an 80 kg adult human. However, Pb is approaching the United States EPA drinking water standard of 15 µg/L and below but approaching the WHO limit of 12.5 µg/day. However, it is important to note that absorption of the inorganic nutrients can strongly vary by age, sex, and how the wine was consumed (i.e., with other foods and their concentrations of potentially competing inorganic nutrients) can decrease gastroenterological absorption and uptake of metals from wine [54]. As prime examples, alcohol in wine was shown to decrease absorption of Ca, Mg, and P [55,56], thus the elements may be present but may not be retained or absorbed by humans. Thus, these values are informational about the potential to supply inorganic nutrients to humans from a nutritional stand point and must not be used for medicinal purposes due to the variability in absorption and uptake.

## 4. Conclusions

The primary objective of this study was to quantify the accumulation and transfer of macronutrients (Ca, K, and Mg), micronutrients (Fe, Mn, Cu, and Zn), and toxic elements (As, Cd, and Pb) from soil to vine to grape berries to wine in vineyards on contrasting lithologies and climates. Our study showed contrasting macro and micronutrient concentrations in soils in varying climates ranging from the Mediterranean climate of southern California to the cold temperate climate of the northeastern U.S. to the subtropical U.S. Furthermore, we found that vines, leaves, and grapes had many significantly different concentrations of macro and micronutrients among the vineyards. However, the differences in vines, leaves, and grape berries generally did not correspond with soil concentrations and we hypothesize that regulation by soil properties, climate, plant ecophysiology and soil management practices controlled uptake and assimilation of nutrients and toxic elements within the grape plants.

The results from studying the white wines show that soil concentrations generally did not correspond with higher nutrient, micronutrient, or toxic metals. Wines from the five vineyards studied had comparable nutrient, micronutrient, and toxic metal concentrations as wines from Germany, Italy, Portugal, Spain, Croatia, Czech Republic, and Japan. An important note is that toxic elements were below concentrations set for drinking water, but they are non-trivial, with As and Pb approaching WHO and USEPA drinking water standards. When considering bioconcentration factors for soil to grape berry transfer, we found that macronutrients are actively concentrated in the berries while Fe, Mn, Cu, Zn, and Pb were generally not accumulated in our study or in previous studies. However, As and Cd were not accumulated nor discriminated against, with BF values close to 0. There are few studies that have reported both As and Cd concentrations in soils and berries, thus this finding is novel and needs further studies to substantiate this effect. The transfer of nutrients and toxic elements from grape berries to wine was studied using a transfer coefficient, which indicated that only Ca, K, and Mg were transferred at a rate suggesting accumulation or addition to the final wine product. All other micronutrients and toxic elements were somewhat to extensively diminished in their concentration. Thus, there appears to be a substantial effect on the geochemistry of the wine from the grape from either the fermentation process, removal by flocculation and clarification, or a dilution effect. A mass-balance approach or application of stable isotopes is necessary to determine if the exact provenance of nutrients and trace elements in wine. Instead, the fermentation process and treatment of the grapes to bottle wine leaves a strong impact on the elemental composition. The abundance of nutrient and toxic elements in wine has potential impacts for human nutrition, as wine can meet or exceed the recommended dietary allowance of Ca, K, Mg, and Fe, depending on the wine and the human consuming it. Moreover, As and Pb are below concentrations and doses that can negatively impact human health but are non-trivial. We conclude that soils, geology, and climate do not appear to generate a unique geochemical terroir as the transfer and concentration of inorganic nutrients appear to be comparable across strongly contrasting vineyards and this has several implications for human health.

## Figures and Tables

**Table 1:** Location and climate for the five vineyards studied.

Vineyard	Location	MAT	MAP	Köppen	Geology	Soil Series †	Grape/Wine
		°C	mm				
V1-NY	Finger Lake Region, NY	8.6	814	Dfb	Sedimentary shale with glacial till	Mardin soil series	Chardonnay
V2-VT	Lake Champlain Region, VT	7.1	850	Dfb	Sedimentary argillite	Covington soil series	Chardonnay
V3-CA1	Temecula Valley, CA	17.5	306	Csa	Granitic alluvium	Greenfield soil series	Chardonnay
V4-CA2	San Diego county, CA	17.1	349	Csa	Granitic alluvium	Visalia soil series	Chardonnay
V5-VA	Shenandoah Valley, VA	13.4	1028	Cfa	Granitic colluvium	Wintergreen Soil series	Chardonnay

† Soil series was obtained from USDA NRCS Web Soil Survey [19].

**Table 2:** Vineyard soil properties. *N* = 9 soil pits were excavated at each vineyard. Standard error values are provided in parentheses.

Vineyard	Soil Series †	Horizon	Depth Interval	pH	% SOM	% Sand	% Silt	% Clay
			cm		%	%	%	%
V1-NY	Mesic Typic	A	0–12	5.97 (0.28)	8.5 (2.1)	33 (6)	55 (8)	12 (3)
	Fragiudepts	B	12–41	5.81 (0.31)	4.6 (1.6)	27 (4)	53 (6)	20 (4)
V2-VT	Mesic Mollic	A	0–22	6.28 (0.27)	10.7 (3.3)	46 (9)	8 (4)	46 (5)
	Endoaqualfs	B	22–32	6.37 (0.24)	8.2 (2.0)	53 (7)	10 (3)	37 (4)
V3-CA1	Thermic Typic	A	0–31	6.23 (0.16)	6.2 (2.4)	45 (7)	41 (5)	15 (3)
	Haploxeralfs	B	31–47	6.32 (0.11)	4.1 (1.1)	48 (6)	40 (5)	12 (3)
V4-CA2	Thermic Typic	A	0–8	6.32 (0.17)	4.3 (1.7)	56 (8)	36 (6)	9 (4)
	Xeropsamments	B	8–31	6.26 (0.14)	2.8 (0.7)	42 (5)	47 (7)	11 (3)
V5-VA	Mesic Typic	A	0–19	5.63 (0.32)	8.5 (2.8)	24 (5)	26 (4)	50 (8)
	Paleudults	B	19–42	5.04 (0.26)	7.2 (2.3)	5 (3)	23 (3)	72 (7)

† Soil series was obtained field observations and from USDA NRCS Web Soil Survey [19].

**Table 3:** Total soil macro and micronutrients for each master horizon at each of the vineyards (*N* = 9 for each master horizon at each vineyard). Standard error values are provided in parentheses.

Vineyard	Material	Ca	K	Mg	Fe	Mn	Cu	Zn	As	Cd	Pb
		g/kg	g/kg	g/kg	g/kg	mg/kg	mg/kg	mg/kg	µg/kg	µg/kg	µg/kg
V1-NY	A horizon	17 (3)	4.5 (0.1)	2.7 (0.2)	7.3 (1.0)	813 (138)	11 (1)	31 (2)	9.0 (1)	0.13 (0.06)	37 (12)
	B horizon	17 (2)	6.8 (0.1)	3.5 (0.2)	7.9 (0.9)	1194 (292)	10 (1)	30 (3)	12 (3)	0.84 (0.07)	52 (13)
V2-VT	A horizon	26 (8)	1.8 (0.3)	2.2 (0.4)	7.1 (2.3)	592 (86)	23 (3)	59 (5)	18 (9)	0.16 (0.05)	2.5 (1.7)
	B horizon	49 (14)	4.3 (0.2)	3.3 (0.1)	7.7 (1.2)	638 (79)	24 (2)	55 (7)	7.4 (4)	0.15 (0.11)	4.0 (0.1)
V3-CA1	A horizon	12 (3)	13 (0.2)	6.5 (0.1)	8.9 (0.2)	577 (13)	26 (4)	161 (12)	4.1 (1.2)	0.05 (0.00)	1.1 (0.1)
	B horizon	14 (2)	12 (0.2)	7.5 (0.2)	8.8 (0.5)	664 (11)	28 (1)	180 (18)	12 (4)	0.07 (0.01)	1.2 (0.1)
V4-CA2	A horizon	12 (4)	2.2 (0.1)	4.7 (0.0)	6.9 (0.2)	513 (7)	37 (4)	164 (17)	7.0 (1.5)	0.08 (0.01)	1.4 (0.1)
	B horizon	11 (2)	2.7 (0.2)	5.9 (0.2)	7.7 (0.8)	509 (17)	37 (3)	162 (14)	5.1 (1.0)	0.06 (0.00)	1.3 (0.1)
V5-VA	A horizon	0.8 (0.0)	0.3 (0.0)	0.3 (0.0)	30 (1.2)	199 (14)	4.5 (0.4)	66 (7)	7.1 (0.5)	0.05 (0.00)	9.2 (0.2)
	B horizon	0.3 (0.0)	0.6 (0.0)	0.3 (0.0)	34 (2.2)	225 (11)	4.1 (0.2)	64 (6)	5.0 (0.4)	0.04 (0.00)	6.5 (0.3)

**Table 4:** Plant macro and micronutrients in vine canes, late season leaves, and harvest-ready grapes at each of the five vineyards. *N* = 18 plants sampled for 3 vine cane segments, 6 to 8 leaves, and 3 grape berry bunches from 18 plants at each of the vineyards. Standard error values are provided in parentheses.

Vineyard	Material	Ca	K	Mg	Fe	Mn	Cu	Zn	As	Cd	Pb
		g/kg	g/kg	g/kg	g/kg	mg/kg	mg/kg	mg/kg	µg/kg	µg/kg	µg/kg
V1-NY	Vine	2.0 (0.8)	6.7 (2.5)	1.9 (0.8)	0.05 (0.02)	2.2 (0.5)	0.3 (0.0)	0.03 (0.01)	0.3 (0.1)	0.02 (0.01)	0.26 (0.07)
V2-VT	Vine	2.3 (0.2)	2.1 (0.4)	0.6 (0.1)	0.01 (0.00)	2.3 (0.3)	3.4 (0.7)	16 (7)	7.2 (1.4)	0.07 (0.03)	0.38 (0.08)
V3-CA1	Vine	4.7 (0.2)	0.1 (0.0)	0.8 (0.1)	0.35 (0.16)	19 (4)	2.7 (0.9)	13 (4)	4.5 (1.1)	0.06 (0.01)	0.08 (0.02)
V4-CA2	Vine	4.0 (0.5)	0.7 (0.3)	0.9 (0.2)	0.59 (0.20)	21 (8)	2.6 (0.3)	23 (8)	8.5 (1.2)	0.10 (0.03)	0.14 (0.03)
V5-VA	Vine	4.6 (0.6)	3.5 (0.8)	2.1 (0.5)	0.09 (0.03)	57 (9)	5.8 (0.7)	80 (21)	8.8 (1.9)	0.08 (0.01)	0.14 (0.02)
V1-NY	Leaves	12 (4)	14 (4)	2.4 (0.8)	0.10 (0.04)	2.7 (1.0)	1.0 (0.3)	0.02 (0.01)	1.2 (0.3)	0.04 (0.01)	0.47 (0.12)
V2-VT	Leaves	15 (3)	1.2 (0.8)	1.2 (0.4)	0.04 (0.01)	8.5 (3.2)	1.7 (0.7)	25 (9)	56 (16)	0.20 (0.07)	0.79 (0.18)
V3-CA1	Leaves	26 (3)	0.1 (0.0)	1.5 (0.3)	1.20 (0.17)	66 (10)	1.4 (0.2)	22 (6)	63 (7)	0.17 (0.03)	0.18 (0.03)
V4-CA2	Leaves	29 (4)	0.1 (0.0)	1.3 (0.2)	1.16 (0.29)	54 (3)	1.1 (0.0)	19 (5)	34 (2)	0.21 (0.04)	0.25 (0.05)
V5-VA	Leaves	5.1 (1.1)	0.4 (0.1)	1.4 (0.3)	0.27 (0.11)	334 (25)	5.6 (1.2)	98 (29)	6.4 (0.4)	0.07 (0.02)	0.16 (0.03)
V1-NY	Grape berry	0.3 (0.2)	10 (3)	0.4 (0.1)	0.04 (0.01)	1.1 (0.3)	0.4 (0.0)	0.01 (0.00)	0.9 (0.1)	0.03 (0.01)	0.34 (0.07)
V2-VT	Grape berry	0.3 (0.1)	0.9 (0.3)	0.2 (0.0)	0.02 (0.01)	3.7 (0.3)	0.7 (0.3)	10 (5)	25 (6.4)	0.13 (0.05)	0.57 (0.19)
V3-CA1	Grape berry	3.1 (0.1)	1.8 (0.3)	0.6 (0.1)	0.95 (0.06)	20 (2)	3.0 (0.7)	4.9 (1.2)	8.1 (0.7)	0.09 (0.01)	0.25 (0.05)
V4-CA2	Grape berry	3.5 (0.3)	4.3 (0.4)	1.0 (0.1)	0.65 (0.24)	21 (6)	2.7 (0.3)	8.4 (1.5)	6.2 (0.6)	0.09 (0.02)	0.21 (0.06)
V5-VA	Grape berry	0.1 (0.0)	0.3 (0.1)	0.2 (0.0)	0.12 (0.04)	26 (9)	2.4 (0.5)	38 (12)	5.0 (0.5)	0.04 (0.01)	0.11 (0.02)

**Table 5:** Mean macro and micronutrients and toxic metals in wines made from grapes of the sampled vineyards in this study and reported in previous studies.

Study	Location	Style	Ca	K	Mg	Fe	Mn	Cu	Zn	As	Cd	Pb
			mg/kg	mg/kg	mg/kg	mg/kg	mg/kg	µg/kg	µg/kg	µg/kg	µg/kg	µg/kg
This study, Vineyard 1	NY, USA	White	106	865	102	2.4	1.0	18	133	5.5	0.2	8.6
This study, Vineyard 2	VT, USA	White	78	527	77	0.4	0.7	11	49	4.1	0.1	3.1
This study, Vineyard 3	CA, USA	White	61	1584	118	1.4	1.0	23	328	3.3	0.1	14.6
This study, Vineyard 4	CA, USA	White	84	1186	120	0.7	1.0	20	274	3.8	0.2	3.1
This study, Vineyard 5	VA, USA	White	66	732	102	0.3	2.8	11	549	4.1	0.1	3.1
Castineira et al., 2004	Germany	White	139		86	4.6	1.7	37	1700	5.1		29
Castineira et al., 2004	Germany	White	144		88	4.3	2.0	18	1200	5.1		23
Catarino et al., 2018	Portugal	Red	72		132	1.5	0.96	30	580	3.2		7.8
Catarino et al., 2018	Portugal	Red	98		124	2.2	1.33	690	380	1.8		7.1
Catarino et al., 2018	Portugal	Red	89		114	3.2	2.55	630	590	2.1		14.5
Catarino et al., 2018	Portugal	Red	84		114	0.6	2.12	900	430	1.3		5.6
Cugnetto et al., 2014	Italy	White	600	1400	73		0.27	80	100			
Cugnetto et al., 2014	Italy	White	1100	2000	110		1.5	220	660			
Darva and Minganti 2019	Italy	White	72	780	82	0.7	6.85	90	470	9.4	3	18
Darva and Minganti 2019	Italy	White	76	674	84	0.8	0.71	80	514	9.4	3	20
Gonzálvez et al., 2009	Spain	Red	382	1025	628	5.0	3.0	300	360		130	
Gonzálvez et al., 2009	Spain	Red	47	826	32	2.0	0.9	80	288		50	48
Gonzálvez et al., 2009	Spain	Red	51	741	75	3.0	0.4	70	300		19	
Kment et al., 2005	Czech	Mixed	108	1126	75	2.6	0.93	448	401	7	0.8	67.1
Leder et al., 2015	Croatia	White	84	683	84	2.3	1.1	140	670	4	0.3	49
Leder et al., 2015	Croatia	White	89	656	92	2.7	1.25	180	770	5	0.4	46
Leder et al., 2015	Croatia	White	78	716	74	1.7	1.08	90	510	1	0.2	54
Leder et al., 2015	Croatia	Red	81	1160	93	3.7	1.03	240	370	2	0.6	78
Leder et al., 2015	Croatia	Red	81	1284	83	3.2	1.15	140	260	1	0.4	79
Leder et al., 2015	Croatia	Red	81	1062	102	4.1	0.94	310	440	3	0.8	80
Shimizu et al., 2018	Japan	White	71	1159	78		1.2					10.3
Shimizu et al., 2018	Japan	White	71	1239	81		1.3					3.4
Shimizu et al., 2018	Japan	White	72	887	77		1.5					3.2
Shimizu et al., 2018	Japan	White	68	1199	95		1.3					3.9
Vrček et al., 2011	Croatia	White	74	753	104	3.4	0.63	539	1180	1.6	0.44	7.1
Vrček et al., 2011	Croatia	White	65	856	69	6.9	0.79	79	847	2.3	0.46	1.3
Vrček et al., 2011	Croatia	White	62	1024	81	0.3	0.37	132	590	1.1	0.20	1.2
Vrček et al., 2011	Croatia	White	71	1340	85	1.9	0.70	217	2270	1.7	0.66	2.8
Vrček et al., 2011	Croatia	White	73.8	707	95	3.8	0.80	532	1574	1.5	0.50	5.8
Vrček et al., 2011	Croatia	White	61.4	702	79	408	0.63	203	1500	0.9	0.33	4.5

## Data Availability

The data presented in this study are available on request from the corresponding author. The data are not publicly available due to privacy agreements with vineyard managers.
